# 2023 Acknowledgment of *mBio* Reviewers

**DOI:** 10.1128/mbio.03015-23

**Published:** 2023-12-19

**Authors:** Arturo Casadevall

**Affiliations:** 1 Johns Hopkins Bloomberg School of Public Health, Baltimore, Maryland, USA

## EDITORIAL

On behalf of the editors of *mBio*, I gratefully acknowledge the following members of our inaugural Junior Editorial Board who served as reviewers for the journal in 2023. These dedicated and well-qualified early-career researchers are a welcome addition to our community of reviewers.

Cynthia Adinortey

Daniel Aridgides

Sheyda Azimi

Lori Delorme Banks

Effie E. Bastounis

Joseph M. Boll

Clotilde Bongrand

Jiandong Chen

Ousmane Cisse

Valerie Cortez

Sebastien Crepin

Nsa Dada

Sophie E. Darch

Nicole J. De Nisco

Stephen K. Dolan

Sarah M. Doore

Elizabeth Draganova

Alison J. Eastman

Melissa Ellermann

Alexandre Gouzy

Melinda Rose Grosser

Asiya Gusa

Andrzej Harris

Tania Henriquez

Saheed Imam

Jayhun Lee

Rebeccah S. Lijek

Rogério Lourenço

Brian Michael Luna

Bruno Martorelli Di Genova

Renata C. Matos

Daniel P. Miller

Angela Marie Mitchell

Roberto Carlos Molina-Quiroz

Irina Novikova

Francisca Nwaokorie

Ceren Ozkul

Kathryn Patras

Atmika Paudel

Diana P. Pires

Diana M. Proctor

Peter W. Ramirez

Paulami Rudra

Daniel Salamango

Lennart Schada Von Borzyskowski

Anna Serquina

Cecilia Alejandra Silva-Valenzuela

Om Prakash Singh

Sanhita Sinharay

Liku B. Tezera

Zulema Udaondo Dominguez

Rachel Van Duyne

Daniel Velez-Ramirez

Julia Willett

Jason H. Yang

Wenqi Yu

Zhengzhong Zou

On behalf of the editors of *mBio*, I gratefully acknowledge the following individuals, who served as reviewers for the journal in 2023. Their time and effort in reviewing articles have been essential in ensuring the high quality of our publications, and their help is greatly appreciated.

Kjersti Aagaard

Stephen T. Abedon

Mohamed A. Abouelkhair

Rachy Abraham

Sabrina Absalon

Michael C. Abt

Hans Ackerman

Brian D. Ackley

Mark D. Adams

Zach Adelman

Vinayak Agarwal

Surya D. Aggarwal

Jesus Aguirre

Tero Ahola

Christopher Aiken

Shin-Ichi Aizawa

Alexandre Alanio

Reem Mohammed Alariqi

Sonja-Verena Albers

John F. Alcorn

Gajender Aleti

Gladys Alexandre

Holly M. Scott Algood

Nezar Al-Hebshi

Matthew Aliota

Sarah M. Allard

Caitilyn Allen

Lee-Ann H. Allen

Luke P. Allsopp

Fadi G. Alnaji

Francis Alonzo

Suhaila Al-Sheboul

Craig Altier

John A. Altin

Shoshy Altuvia

Kishore R. Alugupalli

Pedro M. Alzari

Maximiliano J. Amenabar

Jorge Amich

Karthik Anantharaman

Dohlman Anders

Kirk E. Anderson

Matthew Zack Anderson

Dan Andersson

David R. Andes

Laura Maria Andrade de Oliveira

Sarah A. Andrews

Ekaterina P. Andrianova

Elliot Androphy

Sergio O. Angel

Davide Angeletti

Anastasia Antoniadou

Ruslan Aphasizhev

Rafael Araos

Irina Artsimovitch

Gemma C. Atkinson

Victoria Auerbuch

Benjamin Auxier

Herwig Bachmann

Brian D. Badgley

Yong-Sun Bahn

Guangchun Bai

Dalan Bailey

Michael T. Bailey

Goran Bajic

Susan C. Baker

Siddharth Balachandran

Roberto Balbontín

Hanna-Mari Baldauf

Mitchell F. Balish

Elizabeth Ballou

Bruce W. Banfield

Gert Bange

James D. Bangs

Mukul Bansal

Abhisheka Bansal

Fernando Baquero

Ravi Barabote

Jean-Christophe Barale

Daniel L. Barber

Wendy S. Barclay

Francisco Barona Gómez

Rodolphe Barrangou

Frederic J. Barras

Richard Bartfai

Markus Basan

Philippe Bastin

Tilman Baumstark

Arnold Bayer

Kenneth W. Bayles

Ana Beatriz Ferreira

David H. Bechhofer

Morgan Beeby

James G. Beeson

Joel G. Belasco

Brittany Belin

Boris R. Belitsky

Jose A. Bengoechea

Monsef Benkirane

Megan Bergkessel

Anthony Bertagnolli

Antonio Bertoletti

Sonja M. Best

Sebastien Besteiro

Gira Bhabha

Sumita Bhaduri-Mcintosh

Yuhan Bi

Frederic Bibollet-Ruche

Scott B. Biering

Pablo Bifani

David Bikard

Emanuele G. Biondi

Jordan Bisanz

Jordan E. Bisanz

Karishma Bisht

Biswajit Biswas

Helen Blackwell

Ira J. Blader

David F. Blair

Matthew G. Blango

Jill R. Blankenship

Jesse D. Bloom

Rahul Bodkhe

Dusan Bogunovic

David Bolam

Germán Bonilla-Rosso

Robert A. Bonomo

Kathleen Boris-Lawrie

Arpita Bose

Jeffrey L. Bose

Alberto Bosque-Pardos

Steeve Boulant

David Boulware

Robert B. Bourret

Nicole M. Bouvier

Eric Boyd

Jeff M. Boyd

Jon P. Boyle

Doug E. Brackney

Patricia A Bradford

Axel A Brakhage

Miriam Braunstein

Kelly A. Brayton

Jason Brenchley

Andrew Bridges

Margo A. Brinton

Michael Brockhurst

Nichole A. Broderick

Christopher B. Brooke

James R. Brown

Sam Brown

Edward P. Browne

Petr Broz

John H. Brumell

Andreas Brune

Vincent Michael Bruno

Juliane Bubeck Wardenburg

Susan K. Buchanan

Michael J. Buchmeier

Carmen Buchrieser

Angus Buckling

Nienke Buddelmeijer

Remi Buisson

Silvia Bulgheresi

Vincent Bulone

Lindsey Burcham

James M. Burns

Lori L. Burrows

Nick Burton

Allen R. Buskirk

Victor H. Bustamante

Mark J. Buttner

Mariana X. Byndloss

Matthew T. Cabeen

Sarah Louise Caddy

Curtis Cai

Ben Callahan

Edward M. Campbell

Isaac Cann

David Cánovas

Bin Cao

Delphine Capela

Rey Carabeo

Nicholas H. Carbonetti

Maria de Fátima Cardoso Pereira

Allison F. Carey

Megan E. Carey

Aurelién Carlier

Jason A. Carlyon

Yehuda Carmeli

Jillian Maree Carr

Ronan K. Carroll

Vern B. Carruthers

Agostinho Carvalho

James E. Casanova

Santiago Castillo-Ramírez

Roberto Cattaneo

Colleen M. Cavanaugh

Lynette Cegelski

Debopam Chakrabarti

Romy Chakraborty

Patricia A. Champion

Josephine R. Chandler

Pallavi Chandra

Woo-Suk Chang

Dreux Chappell

John M. Chaston

Ayan Chatterjee

Som S. Chatterjee

Subhadeep Chatterjee

Saurabh Chattopadhyay

Benjamin K. Chen

Hai min Chen

Jiandong Chen

Joseph Chen

Kaiwen Chen

Liang Chen

Qi Chen

Xian-Ming Chen

Zhongzhou Chen

Gong Cheng

Yin-Ru Chiang

W. Seth Childers

Chetan E. Chitnis

Seung-Hyun Cho

You-Hee Cho

Jacob E. Choby

Hyeryun Choe

Zhenlu Chong

Nunya Chotiwan

Mallory J. Choudoir

Graham Christie

David A. Christopher

Hin Chu

Kung-Hui Chu

Nicholas P. Cianciotto

Ousmane H. Cissé

Jan Claesen

Anthony Clark

David J. Clarke

Thomas A. Clarke

Carolina Coelho

Donald M. Coen

Jörn Coers

Rodrigo Cogni

Natalie Cohen

James F. Colbert

James Collins

Alex A. Compton

Andrew Comrie

Ciaran Condon

Brian Conlon

Sarah A. Connolly

John H. Connor

David Cook

Gregory M. Cook

Laura C. Cook

Isabelle Coppens

Stefan Cord-Landwehr

Brendan Cormack

Valerie Cortez

Pierre Cosson

Giulia Costa

Kyle C. Costa

Joseph Cotruvo

Peggy A. Cotter

Alejandro Couce

Mohea Couturier

Timothy L. Cover

Michael M. Cox

Brian Crane

Lindsey B. Crawford

Steven Gaines Cresawn

Daniel Croll

Mark Cronan

Sean Crosson

Timothy Crother

Nicholas J. Croucher

Jun Cui

Elizabeth Culp

Gilberto Curlango-Rivera

Melanie T. Cushion

Daniel M. Czyz

Harry A. Dailey

Johanna Patricia Daily

Fevzi Daldal

Remus Dame

Ajai A. Dandekar

Brian P. Daniels

Pranav Danthi

Chandravanu Dash

Aline M. Da Silva

Atin R. Datta

Dibyadyuti Datta

Christopher Davies

J. Muse Davis

Stephanie Dawes

Daniela De Biase

Zeger Debyser

Luiz Pedro Sorio De Carvalho

Elizabeth De Gaspari

Patrick H. Degnan

Ronnie De Jonge

John P. Dekker

Frank R. Deleo

Maurizio Del Poeta

Diego De Mendoza

Christophe D'Enfert

Yi Zhen Deng

William Depas

Mark Derbyshire

Isabelle Derré

Jeremy P. Derrick

Jigar V. Desai

Mahesh S. Desai

Amedee Des Georges

Aravinda M. De Silva

Jorge De Sousa

Wanderley De Souza

Ulrich Desselberger

Rik L. De Swart

Corrella S. Detweiler

Stephanie Dewitte-Orr

Santosh Dhakal

Suman Kumar Dhar

Thomas Dick

Andreas Diepold

M. Eugenia Dieterle

Stephen P. Diggle

Lubbert Dijkhuizen

Joseph P. Dillard

Huangen Ding

Qiang Ding

Siyuan Ding

Francesca Di Nunzio

Marc S. Dionne

Jocelyne Diruggiero

Dirk P. Dittmer

Roberto Docampo

Christian Doerig

Tobias Doerr

Yohei Doi

Young Do Kwon

Xintong Dong

Xiuzhu Dong

Michael S. Donnenberg

Timothy J. Donohue

Katie J. Doores

Charles J. Dorman

Arnold J. M. Driessen

Christian Drosten

Robert Louis Duda

Breck A. Duerkop

Yann S. Dufour

Nisha K. Duggal

Seána Duggan

Iain G. Duggin

Richard M. Dunham

Jay Dunlap

Paul M. Dunman

Matthew Dunne

Manoj T. Duraisingh

Jeffrey D. Dvorin

Jonathan Dworkin

Anna Earthman

Richard H. Ebright

Mira Edgerton

Martin Egan

Michael Egan

Judith Eisen

Ioannis Eleftherianos

Hiba El Hajj

Irina El Khoury

Craig D. Ellermeier

John Ellis

Courtney K. Ellison

Bert Ely

Iuliana V. Ene

Luis Enjuanes

Alexander W. Ensminger

Emily M. Eriksson

José A. Escudero

Manuel Espinosa

Francisco Estruch

David H. Evans

Matthew J. Evans

Sarah Elisabeth Ewald

Huizhou Fan

Bentley A. Fane

Chuck Shaker Farah

Nina E. Fatouros

Michael J. Federle

Anthony R. Fehr

Michael Feldbrügge

Mario F. Feldman

Zongdi Feng

Andrew K. Fenton

Lucia Fernandez

Dominique Ferrandon

Mariola J. Ferraro

Richard L. Ferrero

Paul D. Fey

Paul L. Fidel

Sergio R. Filipe

Alain Filloux

Cara Fiore

Carolina Firacative

Hannah L. Fischer

Reinhard Fischer

Wolfgang Fischer

Skye R. S. Fishbein

Derek J. Fisher

Matthew C. Fisher

Ferdinando Fiumara

Patrick Flaherty

Derek Fleming

Erik K. Flemington

Trude H. Flo

Ana Flores-Mireles

Laura V. Florez

Daniele Focosi

Alexis Fonseca

Adriana Forero

Elizabeth A. Fortunato

Jarrod R. Fortwendel

Jane Foster

Timothy J. Foster

Enric Frago

Michael T. France

Olivera Francetic

Ashwanth Francis

Carolin Frank

Alan D. Frankel

Eelco Franz

Marnie L. Freckelton

Eric Freed

Eric O. Freed

Daniel E. Freedberg

Megan Culler Freeman

Johannes Freitag

Michal Fried

Elliot S. Friedman

Matthew B. Frieman

Mayuree Fuanghthong

Katsuya Fuchino

Koh Fujinaga

Fumi Fukada

Clay Fuqua

Adam Gaballe

Michaela Ulrike Gack

Jennifer Gaddy

Michael Gaenzle

Marta M. Gaglia

Sara Gago

Cormac G. Gahan

Tom Gallagher

Michael Y. Galperin

Yunn-Hwen Gan

Haichun Gao

George A. Garcia

Mariano A. Garcia-Blanco

José Manuel García-Fernández

Ethan C. Garner

Danielle A. Garsin

Steve George Garvis

Brigitte Gasser

Baoxue Ge

Edward Geisinger

Angie Gelli

Pierre Genevaux

Georgios Germanidis

Aleeza C. Gerstein

Naama Geva-Zatorsky

Benjamin Gewurz

Mahmoud A. Ghannoum

Sourish Ghosh

John G. Gibbons

Tim Gilberger

Stacey D. Gilk

Joseph James Gillespie

Elisa Giovannetti

Mathieu Gissot

Eugene Gladyshev

Erin Gloag

Alain P. Gobert

Adam Godzik

Michael Goettfert

Daniel E. Goldberg

Joanna B. Goldberg

Jonathan Louis Golob

Nardhy Gomez-Lopez

Shane Gonen

Irene Gonzalez

Andrew L. Goodman

Joel M. Goodman

Felicia Goodrum

Isabel Gordo

Santanu Gosh

Heinrich Georg Gottlinger

Caroline Goujon

Celia W. Goulding

Richard L. Gourse

Alexandre Gouzy

Chhabi Kumar Govind

Neil A. Gow

Jeffrey A. Gralnick

Michael D. Grant

Urs F. Greber

Abby Green

Mark S. Gresnigt

Scott S. Grieshaber

Joel S. Griffitts

Anne Grove

Jonathan Grover

Angelika Gründling

Sophie Gryseels

Marc-Jan Gubbels

Randi L. Guest

Karen Guillemin

Allan J. Guimaraes

Karthigayan Gunalan

John S. Gunn

Ju-Tao Guo

Rui Guo

James Gurney

Jenna Guthmiller

Maximiliano Gutierrez

Karen Louisa Haag

Bart L. Haagmans

Hubertus Haas

David Hackstadt

Martin W. Hahn

Matthias Hahn

Peter Halfmann

Patrice P. Hamel

Marie-Louise Hammarskjold

Brian K. Hammer

Tobin Hammer

Sven Hammerschmidt

Linda Hanley-Bowdoin

Blake M. Hanson

Jianjun Hao

Sohei Harada

Matthew J. Harke

Mark Harris

Patrick N. A. Harris

Steven Harris

Dennis J. Hartigan-O'Connor

Amy L. Hartman

Ronald N. Harty

Hassan Hashimi

Theodora Hatziionnou

Vasili Hauryliuk

Tracy H. Hazen

Bin He

Cynthia Y. He

Qiang He

Xuesong He

Ya-Wen He

Lars Hederstedt

Brian P. Hedlund

Dwayne D. Hegedus

Mark T. Heise

Joseph Heitman

Ekaterina E. Heldwein

John D. Helmann

Jeffrey P. Henderson

David R. Hendrixson

Christopher Hernandez

Betsy Herold

Christian Hertweck

Lena J. Heung

Darren E. Higgins

Janet E. Hill

Peter Hill

Markus Hilty

Robert Hirt

Erik Hobbie

Kevin Loren Hockett

Anne G. Hoen

Dirk Hoffmeister

Deborah A. Hogan

James T. Hollibaugh

Kathryn Elizabeth Holt

Dominique Holtappels

Rebekah Honce

Saul M. Honigberg

Alexander R. Horswill

Benjamin Horwitz

Benjamin A. Horwitz

Paul A. Hoskisson

Ya-Ming Hou

Yiping Hou

Kate S. Howell

Jianming Hu

Ke Hu

Wei-Shau Hu

Yangbo Hu

Ying Hu

Jianhua Huang

Juan Huang

Kai Huang

Kerwyn Casey Huang

Lin Huang

Yuefeng Huang

Kai Huang

Lori B. Huberman

Phil Hugenholtz

Adam Joseph Hume

Matthew I. Hutchings

Kevin Hybiske

Jennifer Hyde

Jaekyung Hyun

Michael Ibba

Jose Ibeas

Alexander Idnurm

James Imlay

Francesco Imperi

Roger W. Innes

Thomas R. Ioerger

Gregory C. Ippolito

Satoshi Ishii

Ciro Isidoro

Aymelt Itzen

Tijana Ivanovic

Shingo Iwami

Mary Ann Jabra-Rizk

Joany Jackman

Katrina M. Jackson

William T. Jackson

Marcelo Jacobs-Lorena

Alexander L. Jaffe

Swati Jain

Guilhem Janbon

Hugonnet Jean-Emmanuel

Trushar Jeevan

Kimberly K. Jefferson

Maria Jenckel

Kirk D. C. Jensen

Junhyun Jeon

Sang Eun Jeong

Travis Jewett

Anna Jezierski

Quanjiang Ji

Honglin Jia

Daohong Jiang

Veronica Jimenez

Fernanda Jiménez Otero

Dong-Yan Jin

Chetan Jinadatha

Eric A. Johnson

Clare Jolly

Kristina Jonas

Jeffrey B. Jones

Peter Jorth

Christine Josenhans

Joveeta Joseph

Sarah B. Joseph

Jeffrey Joy

Michael J. Joyner

Praveen Rao Juvvadi

David Kadosh

Jonathan C. Kagan

Michael L. Kahn

Heimel Kai

Maria Kalamvoki

Robert F. Kalejta

Manjula Kalia

Jeremy Phillip Kamil

Jörg Kämper

Bavesh Davandra Kana

Melissa Kane

Timokratis Karamitros

Justin R. Kaspar

Shingo Kato

Rachel A. Katzenellenbogen

Rupinder Kaur

Hangjun Ke

Mary F. Kearney

Daniel B. Kearns

Brandon F. Keele

Jessica L. Keffer

Thomas E. Kehl-Fie

Kylene Kehn-Hall

Lance Edward Keller

Nancy P. Keller

Alyson A. Kelvin

Shannon C. Kenney

Stephen J. Kent

Ayesha Khan

Chang Hyun Khang

Alexander A. Khromykh

Megan R. Kiedrowski

Margaret Kielian

Patricia J. Kiley

Dae-Hyuk Kim

Hwan Keun Kim

Kami Kim

Robert A. Kingsley

Frank Kirchhoff

Ericka Kirkpatrick Roubidoux

Morten Kjos

Sabra Klein

Michael Klemba

Jonas Klingstrom

Andrew Klocko

Leigh Knodler

Laura J. Knoll

James Kobie

Michal Koblizek

Eric Koch

Matthias Koch

Heather Koehler

Arasaki Kohei

Kevin Kohl

Thilo Köhler

Jay Kolls

Masaaki Komatsu

Arash Komeili

James B. Konopka

Eugene V. Koonin

Günther Koraimann

Daniel Kornitzer

Benoit Kornmann

Nicole Marie Koropatkin

Konstantin V. Korotkov

Filip Kovacic

Lukasz Kozubowski

Florian Krammer

Daniel Krappmann

Sven Krappmann

Dave Krause

Bernhard Krautler

Tino Krell

Laurent Kremer

Björn Krenz

Lee Kroos

Laurie T. Krug

Lee R. Krumholz

Damian J. Krysan

Meta Kuehn

Richard J. Kuhn

Ayush Kumar

Priti Kumar

Sanjai Kumar

Barbara N. Kunkel

Stefan Kusch

Simon Labbe

Christian Labenz

Jason T. Ladner

Laimonis A. Laimins

Michael Lalk

Richard J. Lamont

Lena Lampe

Dirk A. Lamprecht

Andrew S. Lang

Michael Lanzer

Alyse Larkin

Joe Larkin

Keren Lasker

Jean-Paul Latge

Amel Latifi

Deric R. Learman

Po-Heng Lee

Amy Lee

Chia Y. Lee

Chung-Young Lee

Jean Claire Lee

Kelly K. Lee

Seok-Yong Lee

Seung Jae Lee

Soo Chan Lee

Vincent T. Lee

Pushkar P. Lele

Stanley M. Lemon

Jose A. Lemos

Sean Leonard

Vanessa Leone

Nancy Leung

Min Levine

Stuart M. Levitz

Gina R. Lewin

Zachary A. Lewis

Bin Li

Chunhao Li

Jianrong Li

Pengfei Li

Bo Liang

Chen Liang

Mary E. Lidstrom

Tami Lieberman

Samuel H. Light

Dominique H. Limoli

Andrew H. Limper

Dandan Lin

Xiaorong Lin

Scott E. Lindner

Jodi A. Lindsay

Michail S. Lionakis

Marc Lipsitch

John Little

Guanqun Liu

Guanshu Liu

Haoping Liu

Huiquan Liu

Luning Liu

Pengfei Liu

Qun Liu

Sanzhen Liu

Weifeng Liu

Wende Liu

Xiang Liu

Yuan Liu

Karen G. Lloyd

Lior Lobel

Shawn R Lockhart

Timothy M. Lohman

Anthony Long

Feng Long

Céline Loot

Allison Lopatkin

Juvenal Lopez

Nora Lopez

Jose L. Lopez-Ribot

Américo Harry López-Yglesias

Michael Lorenz

Stephen Lory

Andrew Lee Lovering

Anice C. Lowen

Catherine Lozupone

Ling Lu

Jun-Bo Luan

Jeremy Luban

Martin Ludlow

Micah A. Luftig

Valeria Lulla

Jennifer M. Lund

Kenneth Lunstrom

Honglin Luo

Min-Hua Luo

Yun Luo

Zhao-Qing Luo

Tania Lupoli

Mor Lurie-Weinberger

Meghan Lybecker

Douglas S. Lyles

Jason Lyu

Mengze Lyu

Bing Ma

Zhe Ma

Benjamin Maasoumy

Erich R. Mackow

Katsumi Maenaka

Suresh Mahalingam

Jennifer Mahony

Benjamin L. Makepeace

Shinji Makino

Fabienne Malagnac

Iran Malavazi

Frank Maldarelli

Iris Maldener

Jan Malinsky

Lara Manganaro

Balaji Manicassamy

Jessica Manning

Sylvie Manuse

Mark Manzano

Yuxin Mao

Emmanouil "Manolis" Maragkakis

Adrian Marchetti

Richard T Marconi

Anthony W. Maresso

Kenneth J. Marians

Luciano A. Marraffini

Pasquale Marrazzo

Ruben Albertus Mars

Gianna Marschmann

David Martinez

Encarnación Martínez-Salas

Luis Martinez-Torres

Adrian Martin-Segura

Tatsunori Masatani

Orietta Massidda

Amy J. Mathers

Ulrike Mathesius

Nicholas J. Matheson

Wendy Maury

Florent Mazel

Shonna M. McBride

Mark S. McClain

Bruce A. McClane

Sarah Mcdonald Esstman

Amelia McGuinness

Peter T. McKenney

James B. McKinlay

Jeffrey Scott McLean

Helen McShane

Jan R. Mead

Markus Meissner

Sahar Melamed

Gregory Melikian

Paola E. Mera

Timothy C. Meredith

Andres Merits

D. Scott Merrell

Dennis Metze

Paul Middleton

Maria Mihaylova

Surabhi Mishra

Rajeev Misra

Aaron P. Mitchell

Patrick Mitchell

Ryo Miyazaki

Valerie Mizrahi

Harry L.T. Mobley

Christopher H. Mody

Bruno Maria Moerschbacher

Mahesh Mohan

Ian Mohr

Wendy W. K. Mok

Michelle Momany

Leila Monjazeb Marvdashti

Caroline Monteil

Andrew J. Monteith

Christopher Montgomery

Cary A. Moody

Robert W. Moon

Hector M. Mora-Montes

Patrice L. Moreau

Benjamin Morehouse

David Morgan

Hiromitsu Moriyama

Minoru Moriyama

Jim Morrissey

Joachim Morschhäuser

Uffe Hasbro Mortensen

Anne Moscona

Gregory William Moseley

Austin Mottola

Jorge Moura De Sousa

W. Scott Moye-Rowley

Patrick Moynihan

Volker Mueller

Monica Mugnier

Conrad W. Mullineaux

Matthew A. Mulvey

Ulrike G. Munderloh

Karl Münger

Jesús Muñoz-Rojas

Carol A. Munro

Christian Münz

Michael Murphy

Thomas Murray

Lawrence C. Myers

Indira U. Mysorekar

Niranjan Nagarajan

Naweed Isaak Naqvi

Muhammad Nawaz

Wilfred Ndifon

Melody N. Neely

Wesley Neely

Oscar A. Negrete

Stuart J. Neil

Daniel C. Nelson

Takayuki K. Nemoto

Sinje Neukirchen

Cécile Neuvéglise

Kathleen M. Neuzil

Michael M. Nevels

Irene L. G. Newton

Mae Newton-Foot

Olivier Neyrolles

Timothy J. Nice

Anthony V. Nicola

Jerome Nigou

Mairi C Noverr

Evgeny Nudler

Joshua J. Obar

Roberta M. O'Connor

Peter Oelschlaeger

Molly Ohainle

Hiroaki Okamoto

Hugo Oliveira

Paulo Oliveira

Antonio Oliver

Michal A. Olszewski

Matthew J. O'Meara

Teresa R. O'Meara

Anders Omsland

Luis Humberto Orellana

Aharon Oren

David A. Ornelles

Kim Orth

Megan Orzalli

Elizabeth Ostrowski

Karen M. Ottemann

Michael Otto

Jeremy George Owen

Clinton R. Paden

Stefano Pagliara

Franco Pagotto

Gustavo F. Palacios

Timothy Palzkill

Laila Pamela Partida-Martinez

John C. Panepinto

Nigel Paneth

Amanda R. Panfil

Kai Papenfort

Hee-Soo Park

Dane Parker

John S. Parker

John S. Parkinson

Colin R. Parrish

Alexander O. Pasternak

Aditya S. Paul

Martin S. Pavelka

Jaclyn Pearson

Kabir Peay

Simone Pecetta

Joao Pedra

Amarendra Pegu

Andrew Pekosz

Philip Pellet

José Penades

Pailan Peng

Joanne Pennock

Daniel R. Perez

Stanley Perlman

Andreas Peschel

Jason M. Peters

Jillian Michelle Petersen

Kevin Pethe

Caitlin Petro

Michaela Petter

Melinda M. Pettigrew

Felicitas Pfeifer

Jennifer Philips

Stacey Piotrowski

Antonio G. Pisabarro

Paul J. Planet

Mircea Podar

Stefan H. Pöhlmann

Norbert Polacek

Alessandra Polissi

Valerie Polonais

Philip Poole

David L. Popham

Michel-Robert Popoff

Peter Preiser

David T. Pride

Richard A. Proctor

Michael J. Pucci

Jose L. Puente

Stefan U. Pukatzki

Lisa A. Purcell

John G. Purdy

Smruti Pushalkar

Guoliang Qian

Zhaohui Qian

Yuan Qiao

Yuqi Qin

Yok-Ai Que

Janet Quinn

Mark Radosevich

Lilliana Radoshevich

Nader Rahimi

Tracy Lyn Raivio

Ricardo Rajsbaum

Stuart A. Ralph

Andoni Ramirez-Garcia

Giordano Rampioni

Matthew Mark Ramsey

Jennifer J. Randall

Tara M. Randis

David Rasko

Philip N. Rather

Sebastian Rausch

Ratna B. Ray

Kasie Raymann

Manjula Reddy

Michael L. Reese

Tiffany A. Reese

R. Keith Reeves

E. Hesper Rego

Alan Rein

Aaron Reinke

Aaron W. Reinke

Olaya Rendueles

Michelle Lynne Reniere

François Renoz

Francis Repoila

Felix A. Rey

Nicole Reynolds

Andrew Rice

Louis B. Rice

Dave Richard

Raúl Riesco Jarrín

Bert K. Rima

Daniel Rinling

Rita Vitorino Moreira Rio

Fabian Rivera-Chávez

Dwayne Roach

Michael D. Robek

Erle S Robertson

Ignasi Roca

Jeremy Rock

Karol Rodríguez-Peña

Alejandra Rodríguez-Verdugo

Roderich Roemhild

Geraint B. Rogers

Jeffrey A. Rollins

Adriana L. Romero-Olivares

Ethan O. Romero-Severson

Severin Ronneau

R. Martin Roop

Roberto Rosales-Reyes

Jason W. Rosch

Miriam A. Rosenbaum

Susan M. Rosenberg

Ilan Rosenshein

Benjamin Ross

Alissa Rothchild

Anna Roujeinikova

Simon Roux

Subhankar Roy-Barman

Daniel Rozen

Natividad Ruiz

Agnieszka Rynda-Apple

Choong-Min Ryu

Claude Sabeta

Ruxana T. Sadikot

Jeroen P. J. Saeij

Jose Saenz

Jason W. Sahl

Adolfo Saiardi

Elke Saile

Kohta Saito

Hassan Salem

Padmini Salgame

Jeanne Salje

María Antonia Sánchez-Romero

Linus Sandegren

Leif Erik Sander

Maria Sandkvist

Lamba Sangare

Dominique Sanglard

Álvaro San Millán

Jorge W. Santo Domingo

Pierre Santucci

Julie Sanville

Sumana Sanyal

Vanessa Sarathy

Christopher Sassetti

Saumya Saurabh

Charles A. Scanga

Dave J. Scanlan

Birgit E. Scharf

Patrick Scheerer

Jeffrey W. Schertzer

Staphan Schilling

Bernhard H. Schink

Susan Schlimpert

Patrick D. Schloss

John Schoggins

Michael Schotsaert

Charles Frank Schuler

Stacey Schultz-Cherry

Thomas F. Schulz

Martin Schuster

Ilan Schwartz

Benjamin Schwessinger

Bianca Sclavi

Jessica A. Scoffield

Barry Scott

Patrick R. Secor

Jessica C. Seeliger

Julie A. Segre

Adnane Sellam

Bert L. Semler

Jonathon W. Senefeld

Anna K. Serquiña

Peter Setlow

William M. Shafer

Yousif Shamoo

Dhanasekaran Shanmugam

Feng Shao

Zongze Shao

Rebecca S. Shapiro

Anupam Sharma

Pushkar Sharma

Amir Sharon

Dana K. Shaw

George M. Shaw

Scarlet S. Shell

Aimee Shen

Bang Shen

Alan Sher

Liang Shi

Pei-Yong Shi

Andrey N. Shkoporov

Maya Shmulevitz

Sarah Short

Deepak Shukla

Carol Sibley

Paul Sigala

Hana Šigutová

Michael J. Sikorski

Anita Sil

Robert F. Siliciano

Sónia Silva

Lynn L. Silver

Cynthia Silveria

Lyle A. Simmons

Francesco Simonetti

Amit Singh

Herman Sintim

Barbara S. Sixt

Eric P. Skaar

Mark S. Smeltzer

Alison G. Smith

Lloyd M. Smith

Terry K. Smith

Kim Sneppen

Evan S. Snitkin

Renee Elizabeth Sockett

Kenneth Söderhäll

Donald Sodora

Erica Sonnenburg

Margherita Sosio

Gerald Spaeth

Konstantin Maria Johannes Sparrer

Paul Spearman

Charles Specht

John David Spencer

Noah Spencer

Pietro Speziale

Tobias Spielmann

Andrew J. Spiers

Katherine R. Spindler

Christina F. Spiropoulou

Adam Mitchell Spivak

Prakash Srinivasan

Martin St. Maurice

Eric V. Stabb

Christina L. Stallings

Alfons J. M. Stams

Spyridon Stavrou

Ida Helene Steen

Natalie Steinel

Joshua Stern

Noam Stern-Ginossar

Frank J. Stewart

Ann M. Stock

Daniel Stoebel

Gisela Storz

Joseph Strauss

Klaus Strebel

Boris Striepen

Jörg Stülke

Bin Su

Chih-Chia Su

Xin-Zhuan Su

Carlos S. Subauste

Agathe Subtil

Christopher P. Suffridge

Sho Sugawara

David J. Sullivan

Guorong Sun

Jianghua Sun

Weina Sun

Yvonne Sun

Mallikarjun Sundaram

George W. Sundin

Way Sung

Mehul S. Suthar

Nobuhiro Suzuki

Sarah Lauren Svensson

Sankar Swaminathan

Michele S. Swanson

Marc Swidergall

Menelaos Symeonides

Kazuo Takayama

Rita Tamayo

Ming Tan

Kai Tang

Yan-Dong Tang

Azuma Taoka

Gregory Tasian

Michelle D. Tate

Gregory Taylor

John W. Taylor

Stephen M. Techtmann

Marcus Melo Teixeira

Paulo José Pereira Lima Teixeira

Benjamin R. Tenoever

Tanel Tenson

Akihiko Terada

Michaela A. Teraves

Scott S. Terhune

Robert Thacker

Ulrich Theopold

Mayuri Vijay Thete

Vinai Chittezham Thomas

Lynmarie K. Thompson

Kai M. Thormann

Rebecca Thurber

Eli Thynne

Leann Tilley

Anna D. Tischler

Daniela Rose Tizabi

Michal Toborek

Stephen Mark Tompkins

Chris J. Tonkin

Bruce E. Torbett

Nathanial Torres

Victor J. Torres

Zsolt Toth

Frances Trail

Cawa Tran

Tuan Minh Tran

Matthew F. Traxler

Massimo Tretiach

Alexandra Trkola

Takafumi Tsuboi

Brian T. Tsuji

Thomas Tu

Elaine I. Tuomanen

Anne-Marie Turner

Kenneth L. Tyler

Kenji Ueda

Stephan Uphoff

Jane Usher

Michael D. Vahey

Eeva Johanna Vainio

Miguel A. Valvano

Bernadette G. Van Den Hoogen

Giel G. Van Dooren

Linda F. Van Dyk

Julia C. Van Kessel

Laurence Van Melderen

Norman Van Rhijn

Jos Van Strijp

Daria Van Tyne

Peter Van Ulsen

Nora Vazquez-Laslop

Andres Vazquez-Torres

Joel Vega-Rodríguez

Daniel E. Vélez-Ramírez

Vittorio Venturi

James Versalovic

Kevin Verstrepen

Ingrid Vetter

Elisa Vicenzi

Jorge E. Vidal

Marisa Vieira De Queiroz

Sumiti Vinayak

Joseph M. Vinetz

Manigandan Lejeune Virapin

Aaron Vogan

Joseph Vogel

Matthew R. Vogt

Benjamin Voisin

Lars Matthias Voll

Waldemar Vollmer

Jay Vornhagen

Joseph Thomas Wade

Matthew K Waldor

Seth T. Walk

Edward Walker

Graham C. Walker

Mark J. Walker

Daniel Wall

David A. Walsh

Jue D. Wang

Linqi Wang

Nian Wang

Pei-Hui Wang

Ping Wang

Qiyao Wang

Wei Wang

Wen-Jun Wang

Wenjing Wang

Zhengyi Wang

Zhibing Wang

Thulasi Warrier

Kenneth Wasmund

Christopher M. Waters

Robert O. Watson

Mary Weber

Lin Wei

Brian C. Weinrick

Susan R. Weiss

Li Wen

Michael R. Wessels

Corey Stephen Westfall

Kelsey Wheeler

Richard Wheeler

Fiona J. Whelan

Sean P. J. Whelan

Judith M. White

Malcolm F. White

Michael W. White

Theodore C. White

Malcolm Whiteway

Jason K. Whitmire

Steven W. Wilhelm

Thomas Williams

Kim C. Williamson

Peter R Williamson

Ian A. Wilson

Patrick Wilson

Richard A. Wilson

Jeffrey Wilusz

Charles Wimpee

Stephen Carlyle Winans

Malcolm E. Winkler

Sebastian E. Winter

Elizabeth Winzeler

Dyann F. Wirth

Stephen Withers

Harald Wodrich

Benjamin E. Wolfe

Lok-Yin Roy Wong

Thomas K. Wood

Robert Woods

Daniel J. Wozniak

Rachel A. F. Wozniak

Daniel Wrapp

Gerard D. Wright

Hui Wu

Qingfa Wu

Ruifang Wu

Song Xiang

Yu Xiao

Jiatao Xie

Xiaoli Xiong

Jiake Xu

Jun Xu

Kai Xu

Chaoyang Xue

Biyun Xue

Srujana Samhita S. Yadavalli

Daisuke Yamane

Masahiro Yamashita

S. Steve Yan

Yuelong Yan

Hang Yang

Jie Yang

Tianyou Yang

George S. Yap

M. N. Frances Yap

Felix Yarovinsky

Gong-Yin Ye

Pamela Yeh

Sowmya Yelamanchili

John Yin

Martina M. Yordanova

Huibin Yu

Xue-Jie Yu

Yueli Yun

Joseph Zackular

Joseph P. Zackular

Sarah Zanders

Xingxing Zang

Raz Zarivach

Egija Zaura

Helen I. Zgurskaya

Jialin Zhang

Jing-Ren Zhang

Lianhui Zhang

Ran Zhang

Tianyu Zhang

Tong Zhang

Xiao-Hua Zhang

Yan Zhang

Yang Zhang

Yu-Zhong Zhang

Zhengguang Zhang

Bo Zhao

Jincun Zhao

Youbao Zhao

Bingyu Zhao

Hui Zheng

Xuhui Zheng

Yong-Hui Zheng

En Min Zhou

Lixin Zhu

Wenhan Zhu

Seth Zost

Shenshen Zou

